# Canopy Temperature and Vegetation Indices from High-Throughput Phenotyping Improve Accuracy of Pedigree and Genomic Selection for Grain Yield in Wheat

**DOI:** 10.1534/g3.116.032888

**Published:** 2016-07-06

**Authors:** Jessica Rutkoski, Jesse Poland, Suchismita Mondal, Enrique Autrique, Lorena González Pérez, José Crossa, Matthew Reynolds, Ravi Singh

**Affiliations:** * International Programs, College of Agriculture and Life Sciences, Cornell University, Ithaca, New York 14853; † Plant Breeding and Genetics Section, School of Integrated Plant Sciences, Cornell University, Ithaca, New York 14853; ‡ Global Wheat Program, International Maize and Wheat Improvement Center (CIMMYT), Ciudad de Mexico, 06600, Mexico; § Department of Plant Pathology, Kansas State University, Manhattan, Kansas 66506

**Keywords:** Secondary traits in genomic selection, GenPred, multivariate analysis, selection index, shared data resource

## Abstract

Genomic selection can be applied prior to phenotyping, enabling shorter breeding cycles and greater rates of genetic gain relative to phenotypic selection. Traits measured using high-throughput phenotyping based on proximal or remote sensing could be useful for improving pedigree and genomic prediction model accuracies for traits not yet possible to phenotype directly. We tested if using aerial measurements of canopy temperature, and green and red normalized difference vegetation index as secondary traits in pedigree and genomic best linear unbiased prediction models could increase accuracy for grain yield in wheat, *Triticum aestivum* L., using 557 lines in five environments. Secondary traits on training and test sets, and grain yield on the training set were modeled as multivariate, and compared to univariate models with grain yield on the training set only. Cross validation accuracies were estimated within and across-environment, with and without replication, and with and without correcting for days to heading. We observed that, within environment, with unreplicated secondary trait data, and without correcting for days to heading, secondary traits increased accuracies for grain yield by 56% in pedigree, and 70% in genomic prediction models, on average. Secondary traits increased accuracy slightly more when replicated, and considerably less when models corrected for days to heading. In across-environment prediction, trends were similar but less consistent. These results show that secondary traits measured in high-throughput could be used in pedigree and genomic prediction to improve accuracy. This approach could improve selection in wheat during early stages if validated in early-generation breeding plots.

Genomic selection (GS) and high-throughput phenotyping (HTP) have great potential to increase the efficiency of wheat, *Triticum aestivum* L., breeding programs. GS is the use of markers covering the whole genome for selection. With GS, reviewed by Lorenz *et al.* (2011), a training set that has been phenotyped and genotyped is used to calibrate a prediction model, which is then used predict the breeding values of a ‘test set’ of genotyped selection candidates. This enables indirect selection for quantitative traits prior to phenotyping. Genomic selection has already been implemented in dairy cattle breeding to increase rates of genetic gain (Pryce and Daetwyler 2012), and simulation studies have demonstrated that GS can increase rates of genetic gain in crop plants (Bernardo and Yu 2007; Wong and Bernardo 2008; Heffner *et al.* 2010). In contrast to GS, HTP is the use of remote and proximal sensing to measure a large number of phenotypes across time and space at low cost and with less labor intensity. If traits measured using HTP are correlated with those of economic importance, HTP data could be used to dramatically increase both the selection accuracy and intensity.

In wheat, canopy temperature (CT) and normalized difference vegetation index (NDVI) are associated with components of grain yield (GY) and can be routinely measured using HTP platforms (Reynolds and Langridge 2016). Canopy temperature is an indicator of evaporative cooling from the canopy surface. Cooler CT is associated with greater stomatal conductance and increased gas exchange rate under irrigated conditions and better hydration status under drought (Pinto *et al.* 2010). Normalized difference vegetation index is an indicator of canopy size and greenness, and is referred to as RNDVI when based on the difference between near-infrared and red reflectance (Wiegand and Richardson 1990a, 1990b), or GNDVI when based on the difference between near-infrared and green light reflectance (Gitelson and Merzlyak 1997). While RNDVI is only sensitive to low chlorophyll-a concentration, GNDVI is sensitive to a wide range of chlorophyll concentrations (Gitelson and Merzlyak 1997). Larger NDVI values are associated with greater biomass accumulation and a faster growth rate when measured during the vegetative (VEG) phase, and a longer grain filling (GF) duration and delayed leaf senescence when measured during the GF phase (Babar *et al.* 2006). Measurements of CT, GNDVI, and RNDVI taken at different growth stages can be considered separate traits, though they generally have a high correlation and can be expected to share some common physiological (Babar *et al.* 2006) and genetic (Pinto *et al.* 2010) bases.

In plant and animal breeding, indirect selection for traits that are expensive or difficult to measure using correlated secondary traits is common. Examples from wheat breeding include selection for reduced plant height to improve harvest index and lodging resistance, and selection for higher protein to improve quality. Selection on secondary traits is advantageous when the secondary trait is highly heritable, highly genetically correlated with the target trait, and it is inexpensive to measure relative to the target trait. One of the challenges of using secondary traits for indirect selection is that the relative utility of secondary traits is situation dependent. Incorporating secondary traits in multivariate pedigree or genomic prediction models partially overcomes this problem because genetic covariances between traits are estimated using a model training set that is representative of the selection candidates and evaluated in the target environment(s). When used in multivariate pedigree or genomic prediction models, secondary traits have been found to improve prediction accuracy and reduce bias compared to univariate models, especially when secondary traits are measured on both the model training population and the selection candidates (Calus and Veerkamp 2011; Jia and Jannink 2012; Pszczola *et al.* 2013). Evidence also suggests that secondary traits, like genome-wide markers, can capture the Mendelian sampling term, and may reduce the advantage of genomic prediction over pedigree prediction (Pszczola *et al.* 2013).

In wheat breeding, prior to GY testing in large replicated plots, a large number of lines are grown in small plots in the field for both seed increase and visual selection. Because measurements of GY on small plots are not meaningful, accurate predictions of GY at this stage would enable an increase in the rate of genetic gain. These predictions could be generated using all available data, including correlated traits and pedigree or genome-wide markers in multivariate mixed models. By including correlated traits observed on selection candidates, both pedigree and genomic selection accuracies could be improved. Because breeding lines must be grown in the field for seed increase prior to GY testing, there would be only a marginal additional cost of HTP, which is quite low, at least in the case of the CIMMYT wheat breeding program.

Canopy temperature, GNDVI, and RNDVI could be excellent secondary traits for pedigree and genomic prediction of GY in wheat because of their high heritabilities and genetic correlations with GY, and because they can be measured remotely on large numbers of selection candidates, possibly during the seed increase generation prior to GY testing. The idea of using CT, GNDVI, or RNDVI to indirectly select for GY is not new; however, an evaluation of these traits for their potential to improve genomic and pedigree prediction accuracies for GY has not yet been done in wheat or other cereals. The objective of this study was to determine if CT, GNDVI, and RNDVI measured using an aerial HTP platform during VEG and GF stages could improve accuracies for GY in wheat when used as secondary traits in multivariate pedigree and genomic prediction models.

## Materials and Methods

### Phenotyping

A total of 1092 inbred breeding lines were grouped into 39 GY trials, and grown during the 2013–2014 crop season at the Norman E. Borlaug Research station in Ciudad Obregon, Sonora, Mexico. Each trial consisted of 28 breeding lines and two checks, and was arranged in an alpha lattice design consisting of three replicates and six blocks. The trials were grown in each of the following environments, ‘early heat’, bed sowing 30 d before the optimal plating date with optimal flood irrigation; ‘optimal’, bed sowing at the optimum planting date with optimal flood irrigation; ‘drought’, bed sowing at the optimum planting date with reduced food irrigation; ‘severe drought’, flat sowing at the optimum plating date with minimal drip irrigation; and ‘late heat’; bed sowing 90 d after the optimal plating date with optimal flood irrigation. Trials in early heat were sown in October. Optimal, drought, and severe drought trials were sown in mid-November, and late heat trials were sown during the last week of February. During the crop season, the optimally irrigated environments received 500 mm of water, and drought and severe drought environments received 250 and 180 mm of irrigation, respectively.

Days to heading (DTHD) was recorded as the number of days from germination until 50% of spike emergence in each plot, and was recorded in the first replicate of each trial. Lodging, which was observed only in the optimal environment, was recorded visually on a zero to five scale. GY was the total plot GY measured after maturity.

High-throughput phenotypic data were collected with a thermal (Model A600 FLIR Infrared camera, Wilsonville, OR) and hyperspectral camera (A-series, Micro-Hyperspec VNIR, Headwall photonics Fitchburg, MA) mounted to a manned aircraft. Data were collected around solar noon time on each date, aligning the aircraft to the solar azimuth for data acquisition. Images of the experimental fields were obtained, and formatted to tabular data by calculating the mean value of the pixels inside the center of each individual trial plot represented as a polygon area on a map.

The 38 cm per pixel CT data were corrected with a linear calibration using parameters calculated based on previous field and camera measurements. For each flight, the individual **i**mages were used to compose a unique mosaic that were then manually georeferenced. The original image data were stored in Kelvin units × 100, and pixel values were converted to Celsius degrees according to the formula (Pixel value)/100 – 273.15.

The 30 cm per pixel hyperspectral data were calibrated radiometrically with coefficients provided by the Laboratory for Research Methods in Quantitative Remote Sensing of the Consejo Superior de Investigaciones Científicas (QuantaLab, IAS-CSIC, Spain) derived with a calibrated uniform light source. Dark frame subtraction was also performed to reduce the noise of the sensor. Correction to decrease the effects of the atmospheric conditions in the images was performed by modeling irradiance based on sun-photometer field measurements (Microtops II, Solar Light Company, Glenside, PA). The images were orthorectified and coarsely georeferenced based on the built-in Inertial Navigation System (INS). For data extraction, images were aligned manually whenever images did not overlay the plot polygons due to INS inaccuracy.

Data from CT, RNDVI, and GNDVI were grouped into VEG and GF stages (Table 1) as in Pask *et al.* (2014) and Mondal *et al.* (2015). There were two to five measurement dates per growth stage for all environments, except late heat, where only one measurement date coincided with the VEG stage. Canopy temperature, GNDVI, and RNDVI were then renamed as CT-VEG, CT-GF, GNDVI-GF, GNDVI-VEG, RNDVI-GF, and RNDVI-VEG according to the growth stage classification.

**Table 1:** Classification of secondary trait measurement dates into vegetative and grain filling stages

Measurement Date	Optimal	Drought	Severe Drought	Late Heat	Early Heat
January 17, 2014	—	VEG	VEG	—	—
January 30, 2014	VEG	VEG	VEG	—	VEG
February 7, 2014	VEG	VEG	—	—	VEG
February 14, 2014	VEG	—	GF	—	VEG
February 19, 2014	VEG	—	GF	—	VEG
February 27, 2014	VEG	GF	GF	—	—
March 11, 2014	GF	GF	GF	—	GF
March 17, 2014	GF	GF	GF	—	GF
March 28, 2014	GF	—	—	—	GF
April 25, 2014	—	—	—	VEG	—
May 21, 2014	—	—	—	GF	—
May 27, 2014	—	—	—	GF	—

Dashes indicate when the measurement date did not coincide with VEG or GF stages and was not used. VEG, vegetative; GF, grain filling.

### Phenotypic data quality control

Within each trial within environment, we calculated repeatability for CT, GNDVI, and RNDVI measured on individual dates, and GY. This within-date repeatability, based on the phenotypic data, was calculated as $r^{2}=\;\frac{\sigma_{g}^{2}}{\sigma_{g}^{2}+\sigma_{\varepsilon }^{2}/nrep}$ where $\sigma_{g}^{2}$ and $\sigma_{\varepsilon }^{2}$ are the genetic, replicate, and residual variances, respectively, and *nrep* is the number of replicates of the breeding lines in the trial (*nrep* = 3). The variance components were estimated by fitting the model:

$$y_{ij}=\;\mu +\;g_{i}+r_{j}+\varepsilon_{ij}\left(\begin{array}{c} i=1\ldots 30\\ j=1\ldots 3 \end{array}\right)$$ (1)

where *y_ij_* is the phenotype, µ is the mean, *g_i_* is the random effect of genotype assuming identical and independently distributed (iid) $g_{i}∼N(0,\sigma_{g}^{2}$), *r_j_* is the random effect of replicate with iid $r_{j}∼N\left(0,\sigma_{r}^{2}\right)$, and *ε_ij_* is the residual with iid $\varepsilon_{ij}∼N\left(0,\sigma_{\varepsilon }^{2}\right)$. Significant outliers (*p*-value < 0.001) were identified using Studentized residuals. If outliers were found, they were removed and repeatability was recalculated.

Similarly, whenever there were multiple measurements across dates we calculated overall repeatability across time and space within each trial for CT-VEG, CT-GF, GNDVI-VEG, GNDVI-GF, RNDVI-VEG, and RNDVI-GF. Overall repeatability for each trait within trial within environment was calculated as $r_{overall}^{2}=\frac{\sigma_{g}^{2}}{\sigma_{g}^{2}+\sigma_{gd}^{2}/ndate+\sigma_{\varepsilon }^{2}/(nrep\;\times ndate)}$ where $\sigma_{g}^{2},\sigma_{gd}^{2},\text{and }\sigma_{\varepsilon }^{2}$ are the genetic, the genotype-by-date interaction, and residual variances respectively, *ndate* is the number of phenotype dates for the trait, which ranged from two to five (Table 1), *nrep* is the number of replicates in the trial (*nrep* = 3). Variance components were estimated by fitting a mixed model similar to (1) but adding the random effects of the dates and the interactions date × environment as:

$$y_{ijk}=\;\mu +g_{i}+d_{j}+r_{k(j)}+gd_{ij}+\varepsilon_{ijk}\left(\begin{array}{c} i=1\ldots 30\\ \begin{array}{c} j=1\ldots ndate\\ k=1\ldots 3 \end{array} \end{array}\right)$$ (2)

where *y_ijk_* is the phenotype, µ is the mean, *g_i_* is the random effect of genotype with iid $\;g_{i}∼N(0,\sigma_{g}^{2}$), *d_j_* is the random effect of date with iid $d_{j}∼N(0,\sigma_{d}^{2})$, *r_k(j)_* is the random effect of replicate nested within date with iid $r_{k(j)}∼N(0,\sigma_{r}^{2})$, *gd_i,j_* is the random effect of genotype-by-date interaction with iid $gd_{ij}∼N(0,\sigma_{gd}^{2})$, and *ε_ijk_* is the residual with iid $\varepsilon_{ijk}∼N\left(0,\sigma_{\varepsilon }^{2}\right)$. If outliers were detected, they were removed, and $r_{overall}^{2}$ was recalculated. We also calculated $r_{overall}^{2}$, excluding individual dates with *r^2^* < 0.01. Individual low-repeatability dates were then removed if doing so improved $r_{overall}^{2}$. Lastly, we removed trials where any of the traits had $r_{overall}^{2}$ < 0.01. Note that, for model (2), we have assumed a very restrictive model that assumes that the dates are identically independent distributed. The resulting dataset (Supplemental Material, File S1) contained 616 lines.

### Genetic value estimation

To estimate genetic values for traits measured across multiple dates for subsequent use in prediction modeling, for each environment, we estimated best linear unbiased estimates (BLUEs) of the breeding lines by fitting the mixed model:

$$y_{ijklm}=\mu +g_{i}+t_{j}+\;d_{k}+r_{l(jk)}+b_{m(jkl)}+\varepsilon_{ijklm}\left(\begin{array}{c} i=1\ldots 616\\ \begin{array}{c} j=1\ldots 22\\ k=1\ldots ndate\\ l=1\ldots 3\\ m=1\ldots 6 \end{array} \end{array}\right)$$ (3)

where *y_ijklm_* is the phenotype, µ is the mean, *g_i_* is the fixed effect of genotype (BLUE), *t_j_* is the random effect of trial with iid $t_{j}∼N(0,\sigma_{t}^{2})$, *d_k_* is the random effect of trait measurement date with iid $\;d_{k}∼N(0,\sigma_{d}^{2})$, *r_l(jk)_* is the random effect of replicate within trial and date with iid $r_{l(jk)}∼N(0,\sigma_{r}^{2})$, *b_m(jkl)_* is the random effect of incomplete block within trial, date, and replicate with iid $b_{m(jkl)}∼N(0,\sigma_{b}^{2})$, and *ε_ijklm_* is the residual with iid $\varepsilon_{ijklm}∼N\left(0,\sigma_{\varepsilon }^{2}\right)$. For traits that were measured only once (*e.g.*, GY), the random effect of date was excluded from model (3). For DTHD, which was measured only once, and on one replicate, the date, replicate, and block effects were excluded from model (3). To enable us to simulate a scenario where the individuals in the test set are not grown in a replicated incomplete block design, as is the case in wheat breeding prior to the yield testing phase, we removed the effects of replicate and incomplete block from model (3), and used this model to estimate BLUEs on a per replicate basis.

To enable across-environment prediction model training, BLUEs were also estimated for each trait across all environments except one which was designated as the validation (testing) environment. To estimate these BLUEs, a random effect for environment was included in model (3), all effects except for the genotype effect were nested within environment, and data from all environments except the validation environment were included in *y*.

For prediction model validation, best linear unbiased predictors (BLUPs) of the breeding lines were calculated for GY within each environment by fitting the mixed model:

$$y_{ijkl}=\mu +g_{i}+t_{j}+r_{k(j)}+b_{l(j,k)}+\beta_{m_{ijkl}}+\varepsilon_{ijkl}\left(\begin{array}{c} i=1\ldots 616\\ j=1\ldots 22\\ k=1\ldots 3\\ l=1\ldots 6 \end{array}\right)$$ (4)

where *y_ijkl_* is the phenotype, µ is the mean, *g_i_* is the random effect of genotype with iid $g_{i}∼N(0,\sigma_{g}^{2})$, *t_j_* is the random effect of trial with iid $t_{j}∼N(0,\sigma_{t}^{2})$, *r_k(j)_* is the random effect of replicate within trial with iid $r_{k(j)}∼N(0,\sigma_{r}^{2})$, *b_l(jk)_* is the random effect of incomplete block within trial and replicate with iid $b_{l(jk)}∼N(0,\sigma_{b}^{2})$, $\beta_{m_{ijkl}}$ is the fixed effect covariate for lodging (fit only in the optimal environment where lodging was observed) for the *i*th genotype in the *j*th trial, *k*th replicate and *l*th block, and *ε_ijkl_* is the residual with iid $\varepsilon_{ijkl}∼N\left(0,\sigma_{\varepsilon }^{2}\right)$. In order to validate prediction models that included a covariate for DTHD, BLUPs for GY corrected for DTHD were calculated by including DTHD as a fixed effect for the *i*th genotype in the *j*th trial in model (4).

### Genotyping

Genotyping-by-sequencing (GBS) (Elshire *et al.* 2011) was used for genome-wide genotyping. All 1092 lines selected for this analysis were included, along with a larger set of 19,965 lines, and were sequenced at 192-plexing on Illumina HiSeq2000 or HiSeq2500 with 1 × 100 bp reads. Single nucleotide polymorphisms (SNPs) were called across all lines using the TASSEL GBS pipeline (Glaubitz *et al.* 2014) anchored to the genome assembly of Chinese Spring (International Wheat Genome Sequencing Consortium 2014).

SNP calls for the set of 1092 breeding lines were extracted, and then markers were filtered so that percent missing data and percent heterozygosity per marker did not exceed 80% and 20%, respectively. Next, lines with > 80% missing marker data were removed, and markers were recoded as –1, 0, and 1, corresponding to homozygous for the minor allele, heterozygous, and homozygous for the major allele respectively. Lines that did not overlap with the set of 616 lines with quality phenotypic data for all traits in all environments were removed. Next, markers with a minor allele frequency < 0.01, and > 80% missing data were removed, and missing data were imputed with the marker mean. A total of 12,083 SNPs scored on 557 individuals was used for subsequent analysis.

### Pedigree and genomic relationship matrices

For the set of 557 lines with quality phenotypic and genotypic data, the genomic relationship matrix (File S2) was estimated according to equation 15 in Endelman and Jannink (2012). For the same set of lines, the pedigree relationship matrix (File S3) was estimated as 2× the coefficient of parentage.

### Square roots of heritabililites and genetic correlations

For traits measured across multiple time points, square root of broad sense heritability (phenotypic selection accuracy) on a single plot basis, $H_{plot\;}$, and on a line mean basis, $H_{line\;\;}$, for each trait within environment were calculated as $H_{plot}=\sqrt{\frac{\sigma_{g}^{2}}{\sigma_{g}^{2}+\sigma_{gd}^{2}/ndate+\sigma_{\varepsilon }^{2}/ndate}}$, and $H_{line}=\sqrt{\frac{\sigma_{g}^{2}}{\sigma_{g}^{2}+\sigma_{gd}^{2}/ndate+\sigma_{\varepsilon }^{2}/(nrep\times ndate)}}$, where $\sigma_{g}^{2}$ is the genetic variance, $\sigma_{\varepsilon }^{2}$ is the error variance, $\sigma_{gd}^{2}$ is the genotype-by-date interaction variance, *ndate* is the number of measurement dates for the trait, and *nrep* is the number of replications (*nrep* = 3). A random effect of genotype-by-date interaction effect was added to model (3) for variance component estimation. Square roots of heritabilities were also estimated for all traits corrected for DTHD by adding a fixed effect covariate for DTHD to the model. For traits that were measured at one point in time (such as GY), $H_{plot\;}$and, $H_{line}$, were calculated as $H_{plot}=\sqrt{\frac{\sigma_{g}^{2}}{\sigma_{g}^{2}+\sigma_{\varepsilon }^{2}}}\;$ and $H_{line}=\sqrt{\frac{\sigma_{g}^{2}}{\sigma_{g}^{2}+\sigma_{\varepsilon }^{2}/nrep}}$, and for variance component estimation, the effect of measurement date and genotype-by-date interaction was removed from the model.

Genetic correlations between traits including CT-VEG, CT-GF, GNDVI-VEG, GNDVI-GF, RNDVI-VEG, RNDVI-GF, and GY were calculated based on estimates of variances and covariances from a multivariate Gaussian mixed model:

$$\left[\begin{array}{c} y_{1}\\ \vdots \\ y_{n} \end{array}\right]=\left[\begin{array}{cc} X & 0\\ \vdots & \vdots \\ 0 & X \end{array}\right]\left[\begin{array}{c} u_{1}\\ \vdots \\ u_{n} \end{array}\right]+\left[\begin{array}{cc} Z & 0\\ \vdots & \vdots \\ 0 & Z_{n} \end{array}\right]\left[\begin{array}{c} a_{1}\\ \vdots \\ a_{n} \end{array}\right]+\left[\begin{array}{c} \varepsilon_{1}\\ \vdots \\ \varepsilon_{n} \end{array}\right]$$ (5)

where *n* is the number of traits, y_1_ is a vector of BLUEs for trait one, y_n_ is a vector of BLUEs for trait *n*, where BLUEs were estimated using model (3), **X** is the fixed effects design matrix, which is the same for each trait, **u**_1_ is a vector of fixed effects for trait one, **u**_n_ is a vector of fixed effects for trait n, **Z** is the random effects design matrix, which is the same for each trait, **a**_1_ is a vector of fixed effects for trait one, **a**_n_ is a vector of fixed effects for trait n, ε_1_ is a vector of residuals of trait one, and **ε_n_** is a vector of residuals for trait n. Variance components were estimated assuming $\left[\begin{array}{c} a_{1}\\ \vdots \\ a_{\text{n}} \end{array}\right]∼N(0,A⊗H)$, and again assuming $\left[\begin{array}{c} a_{1}\\ \vdots \\ a_{\text{n}} \end{array}\right]∼N(0,G⊗H)$, where **A** is the pedigree relationship matrix, **G** is the genomic relationship matrix, and **H** is the variance-covariance matrix for the breeding values of the traits. In both models, $\left[\begin{array}{c} \varepsilon_{1}\\ \vdots \\ \varepsilon_{\text{n}} \end{array}\right]∼N(0,I⊗R)$, where **I** is an identity matrix, and **R** is residual variance-covariance matrix between the traits. The covariance matrices **H** and **R** were assumed unstructured. For each relationship matrix, **A** and **G**, correlations were estimated twice, once including a fixed effect covariate for lodging in the optimal environment, and once including a fixed effect covariate for DTHD in all environments and lodging in the optimal environment. To estimate the genetic correlations between DTHD and GY in each environment, and with each relationship matrix, we used bivariate models (model (5), with *n* = 2 traits).

### Prediction models and validation

**Figure 1:**
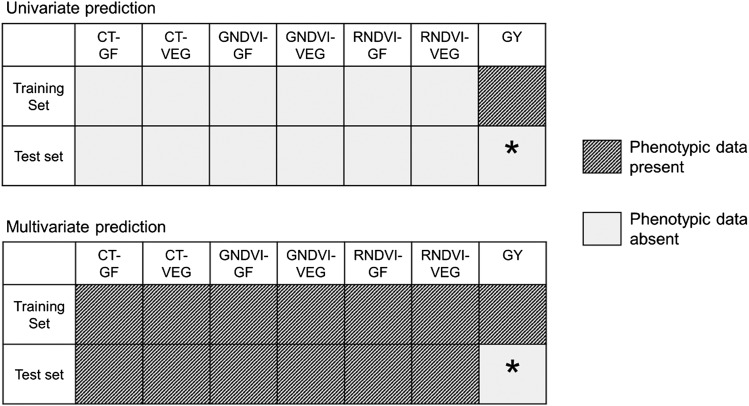
Data used for univariate and multivariate prediction modeling. Each box indicates the presence or absence of phenotypic data for a particular trait on either the training or test set. Presence and absence of phenotypic data are indicated by black stripes and solid gray, respectively. The trait and population of interest for prediction is marked with a black asterisk. The traits include CT during the grain filling phase (CT-GF), CT during vegetative phase (CT-VEG), GNDVI during the grain filling phase (GNDVI-GF), GNDVI during the vegetative phase (GNDVI-VEG), RNDVI during the grain filling phase (RNDVI-GF), RNDVI during the vegetative phase (RNDVI-VEG), and grain yield (GY).

After quality control, complete data on 557 breeding lines remained, and was used for prediction modeling. Accuracies from a multivariate mixed model incorporating GY on the training set only, and secondary traits on both the training and test sets (Figure 1) were compared to those of a univariate mixed model, which does not incorporate secondary traits. The univariate prediction model was:

$$y_{i}=\mu +\beta_{m_{i}}+g_{i}+\varepsilon_{i}$$ (6)

where *y_i_* are the BLUEs of the breeding lines from model (3), µ is the mean, $\beta_{m_{i}}$is a fixed effect covariate for lodging fit only in the optimal environment, *g_i_* is a random effect of genotype with $g_{i}∼N(0,G\sigma_{g}^{2})$ or $g_{i}∼N(0,A\sigma_{g}^{2})$, and *ε_i_* is the residual error with iid $\varepsilon_{i}∼N\left(0,\sigma_{\varepsilon }^{2}\right)$.

The multivariate prediction models were as described in model (5), and assumed either $\left[\begin{array}{c} a_{1}\\ \vdots \\ a_{\text{n}} \end{array}\right]∼N(0,A⊗H)$ or $\left[\begin{array}{c} a_{1}\\ \vdots \\ a_{\text{n}} \end{array}\right]∼N(0,G⊗H)$. Prediction models included a covariate for lodging in the optimal environment. Whenever training and test set secondary trait data were not based on the same number of observations, leading to heterogeneous residual variances, weights based on the number of observations were included in the diagonal of **R**.

Accuracies were estimated using fivefold cross validation with the same fold assignment for all analyses. For each fold, the predictive ability was calculated as the Pearson’s correlation between predictions for GY and the BLUPs for GY from model (4). Prediction accuracy, $r_{g}$, was calculated as $r_{g}=\bar{r_{p}}/H_{line}$, where $\bar{r_{p}}$ is the mean predicative ability across folds. SE of the prediction accuracy ($SE_{{\bar{r}}_{g}}$) was calculated as $SE_{{\bar{r}}_{g}}=\frac{\sigma_{r_{p}}}{H_{line}\times \sqrt{5}}$, where $\sigma_{r_{p}}$ is the SD of the predictive ability. The average accuracy across the five environments was calculated as the mean of the prediction accuracies (with one accuracy per environment). The SE of the average accuracy was calculated as the SD of the accuracies divided by $\sqrt{5}$. Cross validation was conducted within each environment, and across-environment where training and validation set data were from different environments. To perform the latter, for each environment, we performed fivefold cross validation but with data from the environment of interest left out of the training set.

Multivariate prediction model accuracies were estimated once where BLUEs of secondary traits on the test set included data from all three replicates, and once where BLUEs of secondary traits on the test set were estimated using data from the first replicate only. Accuracies were also estimated including a covariate for DTHD in the prediction models.

To estimate the prediction accuracy for the case where pedigree or genomic relationship information is not available, but phenotypic data for GY is available on the training set and phenotypic data for secondary traits is available on all individuals, we fit the same set of within-environment multivariate prediction models as before, except with **y_1_** and **y**_n_ equal to the vectors of BLUEs of the individuals for traits 1 and *n*, respectively, estimated on a per-replicate basis. To correct for the replicate effect, a random effect of replicate nested within trait was included in the multivariate prediction model, and individuals were assumed unrelated with $\left[\begin{array}{c} a_{1}\\ \vdots \\ a_{n} \end{array}\right]∼N(0,I⊗H)$, where **I** is an identity matrix. The predictions resulting from this model were of total genetic values, whereas the predictions from pedigree and genomic selection models were of breeding values.

### Factors affecting accuracy gained from using secondary traits

All genomic and pedigree prediction model accuracies estimated from cross validation within environment were analyzed to assess four factors affecting the accuracy gained from using secondary traits in pedigree and genomic prediction: i. the relationship matrix used in prediction modeling, ii. the environment, iii. the mean magnitude of the genetic correlation,$\bar{\left|r\right|}$, between secondary traits and GY, and vi. the mean square root of the heritability of secondary traits, *$\bar{H}$*, on either a line mean or single plot basis, depending upon whether secondary traits were replicated or not replicated in the prediction model. To estimate the effects and significance of these factors, we fit the linear regression model

$$y_{i}=\mu +r_{i}+m_{ij}+\beta_{h_{ij}}+\beta_{c_{ij}}+\varepsilon_{ij}\left(\begin{array}{c} i=1\ldots 2\\ j=1\ldots 5 \end{array}\right)$$ (7)

where *y_i_* is the multivariate accuracy minus the univariate accuracy for the corresponding univariate model, µ is the mean, *r_i_* is the effect of relationship matrix used in the prediction model, *m_ij_* is the effect of environment, $\beta_{h_{ij}}$ is the effect of $\bar{H}$, and $\beta_{c_{ij}}$is the effect of $\bar{\left|r\right|}$ between secondary traits and GY. The reference levels for relationship matrix and environment were pedigree and optimal, respectively.

### Software

For high-throughput phenotyping, the software ArcMap (ESRI, Redlands, CA) was used to convert images to tabular data, and for manual georeferencing. ImapQ (Alava Ingenieros, Madrid, Spain) was used to correct the CT data with a linear calibration. Autopano Giga (Kolor SARL, France) was used to construct mosaics of multiple images. ENVI software (Excelis VIS, Boulder, CO) was used to convert pixel values to Celsius degrees. HyproQ (Alava Ingenieros, Madrid, Spain) was used to process the hyperspectral data.

The coefficient of parentage was estimated using the ‘browse’ application in the International Crop Information System software package. All other analyses were done in the R programming language and software environment (http://www.r-project.org). The package ‘ASReml-R’ (Gilmour *et al.* 2009) was used to fit univariate mixed models for square root of heritability and repeatability estimation, and to fit multivariate prediction models. The package ‘rrBLUP’ (Endelman 2011) was used to calculate G. The R package ‘EMMREML’ (http://cran.r-project.org/package=EMMREML) was used to fit univaraite genomic prediction models. The regression model assessing factors affecting accuracy was fit using the R package ‘lm’.

### Data availability

The authors state that all data necessary for confirming the conclusions presented in the article are represented fully within the main body and supplemental information of the article.

## Results

### Square roots of broad sense heritabilities and genetic correlations

Values of $H_{plot}$ and $H_{line\;}$(Table 2) were generally high. Values of $H_{line\;}$were on average 10% higher than their corresponding $H_{plot\;}$values. On average, $H_{plot}$ and $H_{line\;}$values were 13% higher when DTHD was not corrected for. Values of $H_{plot}$ and $H_{line\;}$were highest in early heat, and lowest in severe drought.

**Table 2:** Square root of the broad sense heritabilities on a line mean basis, and on a single plot basis

Environment	Trait	Not Corrected for DTHD		Corrected for DTHD	
		Line Mean	Single Plot	Line Mean	Single Plot
Optimal	CT-GF	0.93	0.83	0.92	0.81
	CT-VEG	0.88	0.81	0.82	0.74
	GNDVI-GF	0.97	0.95	0.95	0.9
	GNDVI-VEG	0.95	0.93	0.81	0.74
	GY	0.83	0.66	0.83	0.65
	RNDVI-GF	0.96	0.93	0.94	0.88
	RNDVI-VEG	0.94	0.92	0.76	0.69
Drought	CT-GF	0.77	0.68	0.75	0.66
	CT-VEG	0.93	0.83	0.92	0.83
	GNDVI-GF	0.97	0.94	0.94	0.89
	GNDVI-VEG	0.75	0.71	0.35	0.31
	GY	0.92	0.81	0.89	0.75
	RNDVI-GF	0.97	0.94	0.96	0.91
	RNDVI-VEG	0.87	0.85	0.48	0.44
Severe drought	CT-GF	0.95	0.86	0.94	0.85
	CT-VEG	0.79	0.6	0.79	0.6
	GNDVI-GF	0.98	0.95	0.96	0.9
	GNDVI-VEG	0.53	0.48	0	0
	GY	0.97	0.91	0.93	0.83
	RNDVI-GF	0.97	0.93	0.95	0.9
	RNDVI-VEG	0.82	0.79	0.46	0.42
Late heat	CT-GF	0.81	0.66	0.81	0.65
	CT-VEG	0.76	0.56	0.75	0.54
	GNDVI-GF	0.97	0.93	0.96	0.89
	GNDVI-VEG	0.89	0.75	0.76	0.56
	GY	0.96	0.89	0.96	0.89
	RNDVI-GF	0.87	0.82	0.74	0.67
	RNDVI-VEG	0.95	0.86	0.92	0.8
Early heat	CT-GF	0.96	0.88	0.94	0.85
	CT-VEG	0.91	0.85	0.81	0.71
	GNDVI-GF	0.99	0.97	0.96	0.91
	GNDVI-VEG	0.95	0.92	0.82	0.76
	GY	0.91	0.78	0.86	0.7
	RNDVI-GF	0.98	0.96	0.95	0.88
	RNDVI-VEG	0.98	0.96	0.9	0.84

DTHD, Days to heading; CT, Canopy temperature; GNDVI, Normalized difference vegetation index based on the difference between near-infrared and green light reflectance; GY, Grain yield; RDNVI, Normalized difference vegetation index based on the difference between near-infrared and red reflectance; GF, Grain filling; VEG, Vegetative.

In general, genetic correlations estimated with **A** and **G** were similar; thus, the genetic correlations reported (Table 3, Figure S1, and Figure S2) are an average across estimates using A and G. Correlations ≥|0.09| are significant at the 0.05 level of significance. Genetic correlations between GY and secondary traits (Table 3) ranged in magnitude according to the environment and correction for DTHD. Both with and without correcting for DTHD, genetic correlations were largest in magnitude in early heat and late heat. Secondary trait genetic correlations with GY also ranged in direction depending on the environment. For example, genetic correlation between GNDVI and GY was negative in severe drought, and positive in optimal. Genetic correlations between CT and GY were consistently negative in all environments. Correcting for DTHD almost always reduced the genetic correlations between secondary traits and GY, except for in late heat and optimal. The genetic correlations between GY and DTHD in optimal, drought, severe drought, late heat, and early heat were 0.17, –0.49, –0.71, –0.15, and 0.72, respectively. Genetic correlations between secondary traits varied depending upon the environment (Figure S1 and Figure S2). Drought and severe drought showed a similar pattern of genetic correlations, which was different from that of optimal, late heat, and early heat.

**Table 3:** Genetic correlations between secondary traits and grain yield

Environment	DTHD correction	Secondary Traits					
		CT-GF	CT-VEG	GNDVI-GF	GNDVI-VEG	RNDVI-GF	RNDVI-VEG
Optimal	Uncorrected	−0.65	−0.5	0.27	0.38	0.33	0.33
	Corrected	−0.63	−0.49	0.24	0.44	0.33	0.35
Drought	Uncorrected	−0.59	−0.53	−0.29	−0.41	−0.12	−0.43
	Corrected	−0.59	−0.51	−0.06	−0.23	0.03	−0.42
Severe drought	Uncorrected	−0.41	0.01	−0.54	−0.62	−0.4	−0.77
	Corrected	−0.4	0.03	−0.46	−0.46	−0.33	−0.73
Late heat	Uncorrected	−0.73	−0.66	0.44	−0.14	0.34	0.47
	Corrected	−0.73	−0.65	0.54	−0.13	0.39	0.51
Early heat	Uncorrected	−0.7	−0.71	0.71	0.61	0.73	0.67
	Corrected	−0.7	−0.72	0.68	0.58	0.71	0.67

Values ≥ |0.09| are significant at the 0.05 level of significance. DTHD, Days to heading; CT, Canopy temperature; GNDVI, Normalized difference vegetation index based on the difference between near-infrared and green light reflectance; GY, Grain yield; RDNVI, Normalized difference vegetation index based on the difference between near-infrared and red reflectance; GF, Grain filling; VEG, Vegetative.

### Effect of secondary traits on accuracy within environment

**Figure 2:**
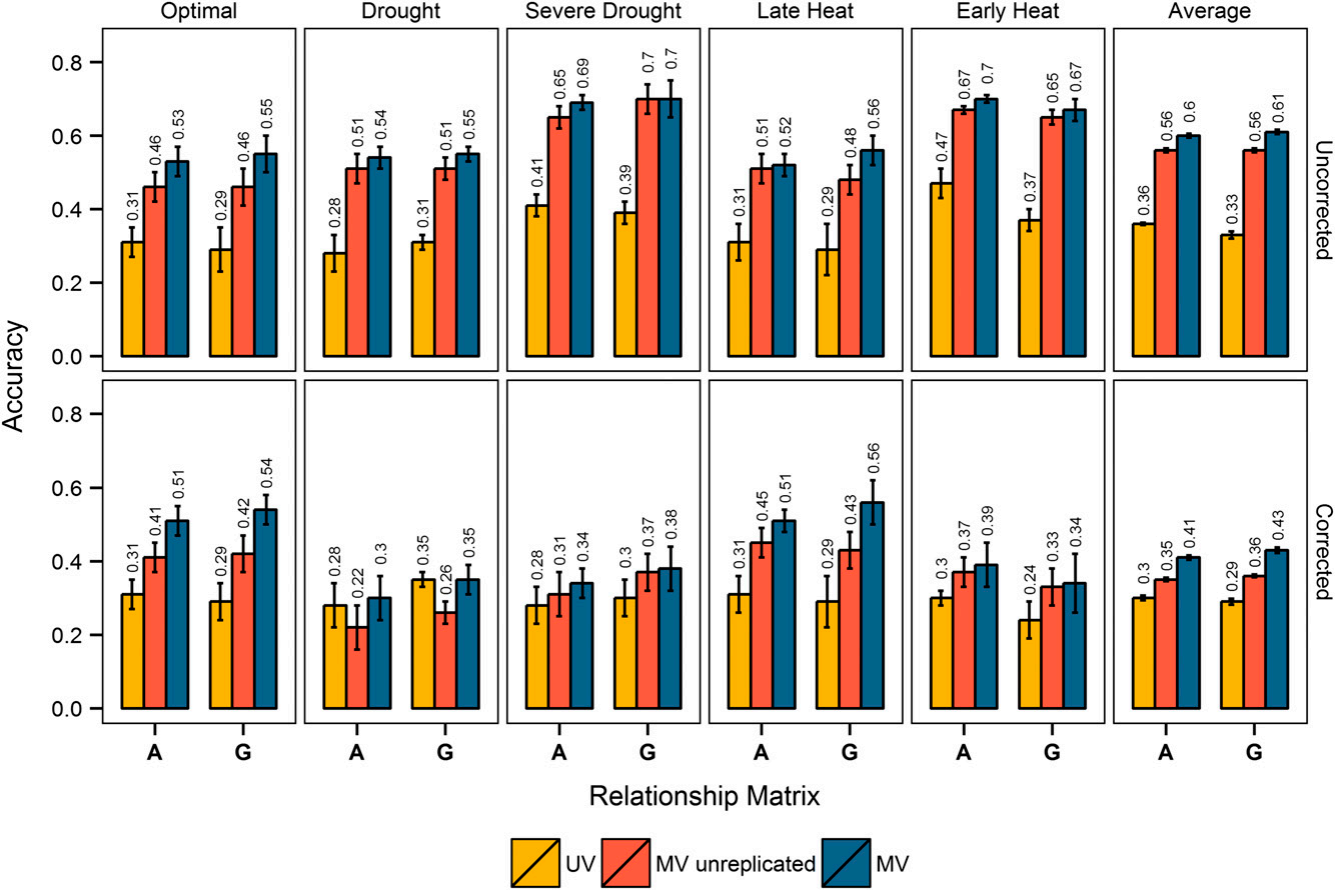
Univariate and multivariate prediction accuracies within environment, with and without correcting for days to heading. Within environment prediction accuracies from models using pedigree (**A**) and genomic (**G**) relationship are shown for each of the five environments, and for the average accuracy across all environments. Yellow, accuracies from univariate models (UV); red, accuracies from multivariate models (MV) where secondary trait data were from one replicate; blue, accuracies from MV models where secondary trait data were from three replicates. The first row of bar plots shows accuracies without correcting for days to heading (uncorrected), and the second row of bar plots shows accuracies with correcting for days to heading (corrected). SE is shown with error bars, and accuracy values are printed above each bar.

Multivariate pedigree and genomic prediction models that incorporated secondary trait data on training and test sets were consistently, and often substantially, more accurate than their corresponding univariate prediction models for GY within environment (Figure 2), demonstrating that secondary traits from HTP can improve pedigree and genomic prediction accuracy. No improvement in accuracy was observed when secondary traits were observed only on the training set (data not shown). The improvement in accuracy due to secondary traits depended on the replication of secondary traits in the test set, and whether we corrected for DTHD.

On average, without correcting for DTHD, secondary traits measured on all three replicates in the test set led to a 67% improvement in pedigree prediction accuracy, and an 85% improvement in genomic prediction accuracy. When we used only one replicate of secondary trait data on the test set, secondary traits lead to a 56% increase in pedigree prediction accuracy, and a 70% increase in genomic prediction accuracy.

When correcting for DTHD, on average, secondary traits improved pedigree prediction accuracy 37%, and genomic prediction accuracy 48%, when secondary trait data on the test set was replicated. When only one replicate of secondary trait data were used on the test set, secondary traits increased pedigree prediction accuracy 17%, and genomic prediction accuracy 24%.

Overall, we observed consistent trends in accuracy. Multivariate prediction models with replicated secondary trait data on the test set were most accurate, followed by multivariate prediction models with unreplicated secondary trait data on the test set, and univariate prediction models. Also, secondary traits had a slightly larger impact on accuracy in the genomic prediction models compared to the pedigree prediction models, and correcting for DTHD consistently reduced accuracies and led to smaller differences in accuracy between the multivariate and univariate models. The largest impact of correcting for DTHD was observed in drought. In drought, without correcting for DTHD, secondary traits lead to the largest percent increase in accuracy compared to all other environments. In contrast, when correcting for DTHD in drought, secondary traits led to no improvement or a decrease in accuracy. The smallest impact of correcting for DTHD was observed in late heat, where the multivariate and univariate prediction accuracies were largely unaffected.

### Effect of secondary traits on across-environment accuracy

**Figure 3:**
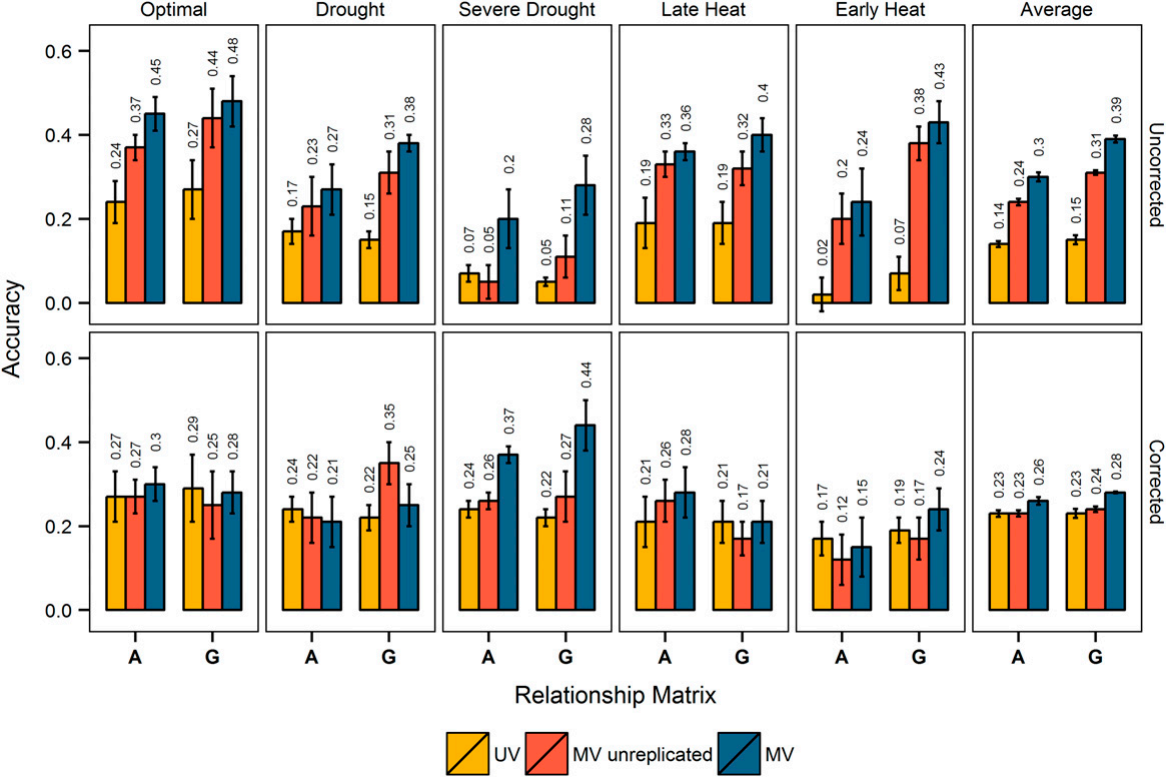
Univariate and multivariate prediction accuracies across-environment, with and without correcting for days to heading. Across-environment prediction accuracies from models using pedigree (**A**) and genomic (**G**) relationship are shown for each of the five environments, and for the average accuracy across all environments. For each environment, the training set used in prediction contained data from all environments except the environment of interest. Yellow, accuracies from univariate models (UV); red, accuracies from multivariate models (MV) where secondary trait data were from one replicate; blue, accuracies from MV models where secondary trait data were from three replicates. The first row of bar plots shows accuracies without correcting for days to heading (uncorrected), and the second row of bar plots shows accuracies with correcting for days to heading (corrected). SE is shown with error bars, and accuracy values are printed above each bar.

Without correcting for DTHD, across-environment multivariate pedigree and genomic prediction models incorporating secondary trait data on training and test sets were consistently, and substantially, more accurate than their corresponding univariate prediction models (Figure 3). On average, secondary traits improved pedigree prediction accuracy 114%, and genomic prediction accuracy 160%, when secondary trait data on the test set was replicated. When only one replicate of secondary trait data on the test set was used, secondary traits improved pedigree prediction accuracy 71%, and genomic prediction accuracy 107%.

When correcting for DTHD, across-environment multivariate pedigree and genomic prediction models were frequently about as accurate as, or less accurate than, their corresponding univariate prediction models (Figure 3). On average, when secondary trait data on the test set was replicated, secondary traits improved pedigree prediction accuracy 13%, and genomic prediction accuracy 22%. When secondary trait data on the test set was not replicated, on average secondary traits did not increase pedigree prediction accuracy, and increased genomic prediction accuracy 4%.

The overall trends for across-environment prediction were that multivariate models were more accurate than univariate models only without correcting for DTHD, secondary traits lead to greater accuracies when secondary trait data were replicated *vs.* not replicated in the test set, and correcting for DTHD increased the univariate prediction accuracies and reduced the gain in accuracy from using secondary traits.

### Prediction accuracies from models assuming lines were unrelated

We found that, on average, when predictor trait data were not replicated on the test set, and when correcting for DTHD, multivariate prediction models that assumed lines were unrelated were either equally accurate or slightly less accurate than genomic and pedigree predictions incorporating secondary traits (Table S1). When secondary trait data were replicated on both training and tests sets, and prediction models did not correct for DTHD, multivariate prediction models that assumed lines were unrelated were actually slightly more accurate on average compared to the multivariate pedigree and genomic prediction models. This indicates that, without estimates of relationship, secondary trait data can be useful for predicting total genetic values of GY.

### Factors affecting accuracy gained from using secondary traits

The regression model that we fit to examine factors affecting the accuracy gained from using secondary traits in pedigree and genomic prediction was significant (*P* << 0.01) with an adjusted *r^2^* = 0.78. Based on this model, we found that the gain in accuracy from using secondary traits was significantly associated with a) $\bar{\left|r\right|}$ between secondary traits and GY (*P* < 0.05), and b) $\bar{H}$ of secondary traits (*P* << 0.01) (Table 4). We also found that compared to the optimal environment, the accuracy gained from using secondary traits was significantly greater in late heat, and early heat (*P* << 0.01).

**Table 4:** Regression model coefficients explaining the accuracy gained from secondary traits

Coefficients*^a^*	Effect	*P*-Value
Intercept	−0.97	1.6 × 10^−9^
Relationship matrix, genomic	−2.8 × 10^−3^	0.74
Environment, drought	1.1 × 10^−2^	0.65
Environment, severe drought	3.8 × 10^−2^	0.23
Environment, late heat	0.12	2 × 10^−7^
Environment, early heat	6.2 × 10^−2^	3.6 × 10^−4^
$\bar{H}$ *^b^* of secondary traits	1.1	2.7 × 10^−10^
$\bar{\left|r\right|}$*^c^* between secondary traits with grain yield	0.57	2.7 × 10^−2^

a The reference level for the factor, relationship matrix is pedigree and the reference level for the factor, environment is optimal.

b *$\bar{H}$*, Mean square root of either line mean or single plot heritability depending upon the replication of the secondary traits in the prediction model.

c *$\bar{\left|r\right|}$*, Mean absolute value of the genetic correlation.

## Discussion

This study found that, in wheat, GNDVI, RNDVI, and CT measured across the crop growth cycle using an aerial HTP platform can substantially improve genomic and pedigree prediction model accuracies for GY when observed on training and test sets and used as secondary traits in multivariate models. Our prediction-model-based approach is an improvement upon conventional indirect selection based on secondary traits for two reasons. First, by using a prediction model, we take advantage of secondary trait data on the selection candidates *per se*, and GY data from selection candidates’ relatives. Second, by using a model training set evaluated for all traits in the target set of environments, secondary traits are appropriately weighted, depending in part upon the magnitude and the direction of their genetic correlations with GY. Because a quality control procedure was used prior to prediction modeling, an important assumption of this study is that there are no major technical errors in the HTP data collection that would give rise to near zero repeatability for trait measurements. We expect this assumption to hold true as HTP data collection and imaging processing pipelines continue to improve, minimizing technical errors.

There was no improvement in accuracy when secondary traits were observed on the training set only, which is consistent with a similar study that evaluated secondary traits for feed intake in cattle (Pszczola *et al.* 2013). This may have been due to the relatively high heritabilities of GY in this study. Based on simulation, including secondary traits only on the training set can improve accuracy when the heritability of the trait of interest is low, and the heritabilities of the secondary traits are high (Jia and Jannink 2012). Thus, in the current study, heritabilities of GY were sufficiently high to make including secondary traits only on the training set ineffective for improving accuracy.

Due to genotype-by-environment interaction, within-environment prediction accuracies were always higher than across-environment prediction accuracies. Thus, comparing the percent increases in accuracy due to secondary traits in across- *vs.* within-environment prediction may be misleading. Based on percent increase in accuracy, without correcting for DTHD, secondary traits appeared to be much more beneficial for prediction across environment than for prediction within environment, when in fact the numerical increases in accuracy due to secondary traits in across-environment prediction were lower than those of within-environment prediction.

Correcting for DTHD improved univariate prediction accuracies across-environment, which suggests that the genotype-by-environment variance for GY corrected for DTHD is lower than that of uncorrected GY. For both within- and across-environment prediction, correcting for DTHD reduced the genetic correlations between GY and the secondary traits, which in turn reduced the accuracy gained from including secondary trait data on the test set. In plant breeding, GY is sometimes corrected for DTHD or another phenological trait in order to avoid indirect response to selection for that trait. To take full advantage of secondary trait data while avoiding indirect selection on a phenological trait, a better approach may be to include data on the phenological trait in a multivariate prediction model along with any available secondary traits, and then use the multivariate BLUPs to calculate a selection index with GY and the phenological trait weighted appropriately.

If pedigree or genomic relationship information are not available, total genetic value for GY can be predicted using secondary trait data, assuming there is a population of lines phenotyped for both GY and secondary traits across multiple replicates that can be used to train a multivariate prediction model. Prediction accuracies for GY genetic value estimated in this way were frequently very similar to prediction accuracies for GY breeding values estimated using multivariate genomic and pedigree prediction models. If we assume that all the genetic variance is additive, this suggests that multivariate pedigree and genomic prediction accuracies were often driven primarily by secondary trait information on the individuals *per se*. However, to ensure the best estimates of breeding values, utilizing pedigree or genomic relationship information is recommended.

The accuracy gained from using secondary traits was associated with $\bar{\left|r\right|}$ between secondary traits and GY, and $\bar{H}$ of secondary traits, which is in agreement with selection index theory. The significance of the effects of late heat and early heat on the accuracy gained from secondary traits is difficult to explain. According to Schaeffer (1984), the greater the absolute difference between the residual and genetic correlations between traits, the greater the percent reduction in the prediction error variance, and therefore greater accuracy, in multivariate BLUP. However, after examining the absolute differences between the residual and genetic correlations in each environment, we still could not explain the significant environment effects. This indicates that the gain in accuracy due to a set of secondary traits may be difficult to predict based only on heritabilities and correlations, and cross-validation in the environments of interest will be required to assess the gain in accuracy from using secondary traits in pedigree and genomic selection.

### Conclusion

We have shown that, in wheat, GY secondary traits measured using an aerial HTP platform can be used to increase pedigree and genomic prediction model accuracies for GY when observed on both the model training and test sets. These prediction models could be useful for making selections in generations where GY cannot be measured accurately but secondary traits can. In crop breeding, there are instances where selection candidates can be phenotyped for some traits, but, due to insufficient seed, high cost of phenotyping, severe weather events, and/or long juvenile phase duration, not all traits of economic importance can be phenotyped. In these cases, traits correlated with the economically important traits could be used in pedigree and genomic prediction models to improve selection accuracy without delaying selection. This approach appears promising in wheat and should be investigated in other crops especially as HTP become more accessible.

Additional research is needed before secondary traits from HTP can be used routinely in wheat breeding to increase pedigree and genomic selection accuracy for GY when GY cannot be measured directly on the selection candidates. First, in this study, we used a simple repeatability model for GNDVI, RNDVI, and CT measurements within a growth stage; however, time series models for measurements of these traits taken across time need to be compared to identify the best model for each trait. This could improve the phenotypic selection accuracy of GNDVI, RNDVI, and CT, potentially leading to better prediction accuracies when these traits are used in pedigree and genomic selection. To help address this need, we are currently working on evaluating repeatability, multivariate, and random regression models for GNDVI, RNDVI, and CT. Second, this study used secondary trait data from large plot sizes suitable for measuring GY, but, for selection, it will be useful to use secondary traits from plots that are of small size due to limited seed availability. Thus, the utility of secondary traits measured using HTP in pedigree and genomic prediction models need to be assessed for the case where the test set individuals are sown in a small plots. Lastly, HTP data analysis pipelines that lead to better data quality and faster speed of processing should continue to be developed.

## Supplementary Material

Supplemental Material
