# Supplementary material for: Canopy Temperature and Vegetation Indices from High-Throughput Phenotyping Improve Accuracy of Pedigree and Genomic Selection for Grain Yield in Wheat
Source: G3 (Bethesda). 2016 Jul 6;6(9):2799–808. doi: 10.1534/g3.116.032888 (PMC5015937; doi:10.1534/g3.116.032888)
Supplement: Supplemental Material [file supp_6_9_2799__index.html]

Canopy Temperature and Vegetation Indices from High-Throughput Phenotyping Improve Accuracy of Pedigree and Genomic Selection for Grain Yield in Wheat — Supplemental Material 

# Canopy Temperature and Vegetation Indices from High-Throughput Phenotyping Improve Accuracy of Pedigree and Genomic Selection for Grain Yield in Wheat

## Supplemental Material for Rutkoski, *et al*, 2016

**Files in this Data Supplement:**

- Figure S1 - Genetic correlations between traits. (.pdf, 142 KB)
- Figure S2 - Genetic correlations between traits corrected for days to heading. (.pdf, 142 KB)
- Table S1 - Multivariate prediction accuracies and standard errors for GYa for different assumptions of relationship between lines. (.pdf, 69 KB)
- File S1 - Plot-level phenotypic data after quality control. The genotype identifier column contains the identification numbers for the 616 lines that remained after quality control. The trial column indicates the trial nested within environment, the replicate column indicates the replicate nested within trial and environment, and the block column indicates the incomplete block nested within replicate, trial and environment. The phenotype date column indicates the date when the phenotypic value was collected in the field. The growth stage column indicates whether the phenotype date coincided with the vegetative (VEG) or grain filling (GF) phase. The trait column indicates the trait that was phenotyped, either canopy temperature (CT), days to heading (DTHD), green normalized difference vegetation index (GNDVI), grain yield (GY), lodging score (LODGING) or red normalized difference vegetation index (RNDVI). The phenotypic value column contains the phenotypic data values on an individual plot basis. Missing values for phenotype date and growth stage are denoted as '-'. (.zip, 1,549 KB)
- File S2 - Genomic relationship matrix for the 557 lines used for prediction modeling. The first row and column contains the identification numbers for the lines. (.zip, 2,594 KB)
- File S3 - Pedigree relationship matrix for the 557 lines used for prediction modeling. The first row and column contains the identification numbers for the lines. (.zip, 369 KB)
