# Supplementary material for: Canopy Temperature and Vegetation Indices from High-Throughput Phenotyping Improve Accuracy of Pedigree and Genomic Selection for Grain Yield in Wheat
Source: G3 (Bethesda). 2016 Jul 6;6(9):2799–808. doi: 10.1534/g3.116.032888 (PMC5015937; doi:10.1534/g3.116.032888)
Supplement: Supplemental Material [file supp_g3.116.032888_FigureS1.pdf]

Figure S1: Genetic correlations between traits

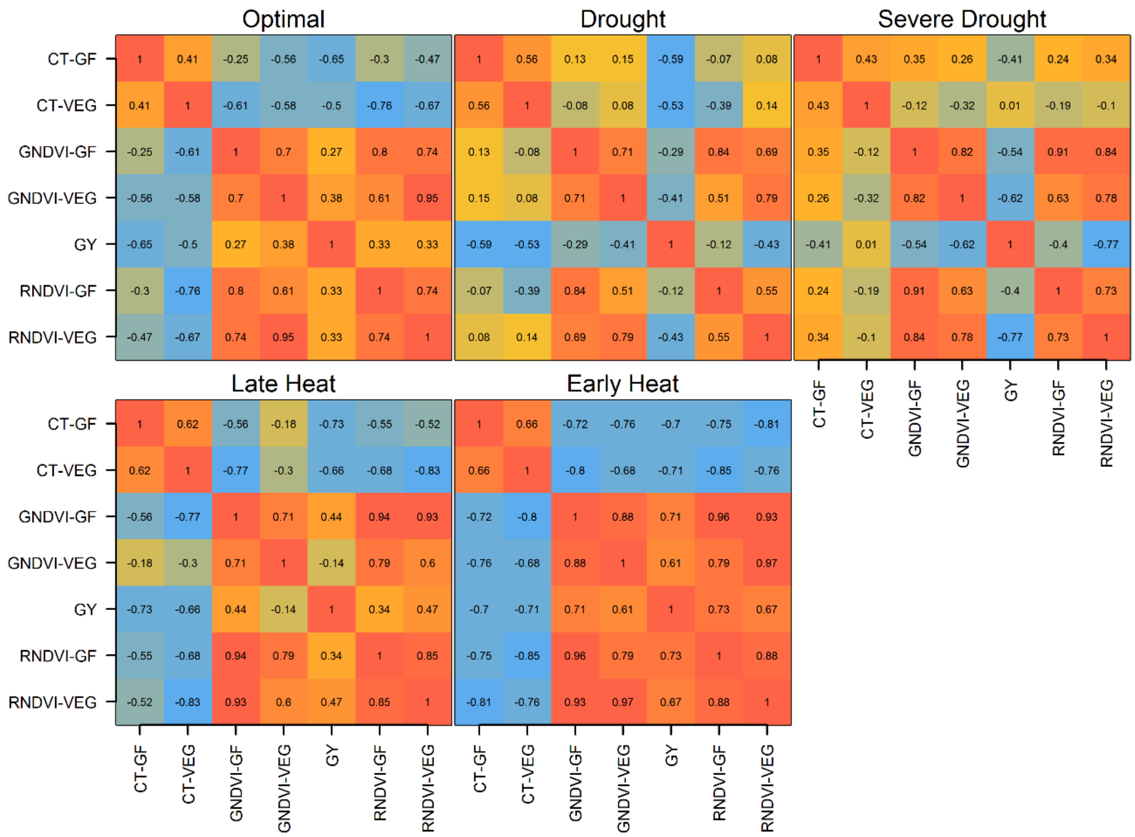

For each environment, genetic correlations between all traits are reported. Large negative and large positive correlations are color coded in shades of blue and red respectively. Intermediate values are shaded in yellow. The genetic correlation shown is the average of the genetic correlations calculated with pedigree and genomic relationship matrices. CT; Canopy temperature, GNDVI; Normalized difference vegetation index based on the difference between near-infrared and green light reflectance, GY; Grain yield, RDNVI; Normalized difference vegetation index based on the difference between near-infrared and red reflectance, GF; Grain filling, VEG; Vegetative. Values greater than or equal to |0.09| are significant at the 0.05 level of significance.
