# Supplementary material for: Canopy Temperature and Vegetation Indices from High-Throughput Phenotyping Improve Accuracy of Pedigree and Genomic Selection for Grain Yield in Wheat
Source: G3 (Bethesda). 2016 Jul 6;6(9):2799–808. doi: 10.1534/g3.116.032888 (PMC5015937; doi:10.1534/g3.116.032888)
Supplement: Supplemental Material [file supp_g3.116.032888_TableS1.pdf]

Table S1: Multivariate prediction accuracies and standard errors for GY[a] for different assumptions of relationship between lines

			Relationship assumption		
DTHD[b] correction	Secondary trait replication	Environment	Unrelated	Pedigree	Genomic
Not corrected	Replicated	Optimal	0.53±0.05	0.53±0.04	0.55±0.05
		Drought	0.61±0.04	0.54±0.03	0.55±0.02
		Severe Drought	0.73±0.03	0.69±0.02	0.7±0.05
		Late Heat	0.59±0.02	0.52±0.03	0.56±0.04
		Early Heat	0.75±0.04	0.7±0.01	0.67±0.03
		Average	0.64±0.04	0.6±0.01	0.61±0.01
	Not replicated	Optimal	0.43±0.05	0.46±0.04	0.46±0.05
		Drought	0.52±0.04	0.51±0.04	0.51±0.03
		Severe Drought	0.69±0.03	0.66±0.03	0.7±0.04
		Late Heat	0.49±0.03	0.51±0.04	0.48±0.04
		Early Heat	0.68±0.04	0.67±0.01	0.65±0.02
		Average	0.56±0.05	0.56±0.01	0.56±0.01
Corrected	Replicated	Optimal	0.5±0.05	0.51±0.04	0.54±0.04
		Drought	0.39±0.09	0.3±0.06	0.35±0.04
		Severe Drought	0.6±0.03	0.34±0.04	0.38±0.06
		Late Heat	0.23±0.05	0.51±0.03	0.56±0.06
		Early Heat	0.31±0.07	0.39±0.06	0.34±0.08
		Average	0.41±0.07	0.41±0.01	0.43±0.01
	Not replicated	Optimal	0.41±0.05	0.41±0.04	0.42±0.04
		Drought	0.29±0.09	0.22±0.06	0.26±0.04
		Severe Drought	0.51±0.02	0.31±0.06	0.37±0.06
		Late Heat	0.15±0.05	0.45±0.04	0.43±0.06
		Early Heat	0.29±0.07	0.37±0.04	0.33±0.08
		Average	0.33±0.06	0.35	0.36

[a]GY; Grain yield, [b]DTHD; Days to heading, [c]Unrelated: covariance between individuals is assumed zero and the predictions are of total genetic values. Pedigree and Genomic; the covariance between individuals is modeled as proportional to pedigree and genomic relationship matrices respectively and the predictions are of breeding values.
